# Annotations

**Published:** 1892-08-20

**Authors:** 


### Annotations.

**Sewing in Schools.**

It is satisfactory to know that, at least in our primary schools, the intellectual development of girls has not been purchased at the cost of neglecting the old and ever-womanly art of needlework. While one is glad to let every child, without distinction of sex, have the chance of wider knowledge of the works of nature and of man, it cannot be denied that in after life an acquaintance with the mysteries of stitching and hemming will be of far more practical value to the majority of "little maids at school" than the most accurate information regarding the war of the Spanish succession, or the formation of glaciers. In one or two schools we find the sewing-machine taught—an innovation which cannot be wholly welcomed if is intended to take the place of instruction in the use of the needle. For not only are there many things in the making of the simplest garment which the machine cannot do, as every practical seamstress will tell you, but in the case of the daughters of the poor you have to allow for the possibility of even the cheapest machine being beyond their purses, and in all cases for the chance of emergencies of time or place where only the most portable *etui* is of practical value. But in many schools the point to which the teaching of sewing is carried is far beyond what is useful or desirable; and themost elaborate efforts are demanded of girls in the higher standards, and of young pupil teachers whose other studies are so exacting that they have little time or energy to give to the marvellous mending and darning which we see exhibited at school inspections. "Fancy work" is rightly discouraged at rate-supported schools, but some of the specimens shown are really a kind of elaborate embroidery. It is one thing to teach girls to do all manner of needlework well and neatly, another to insist on their spending so much time over the mending of a rent that when time has come to be a thing of value to them they will never dream of practising their school methods. The Education Department protests vainly against stitches disproportionately fine to the cloth employed; sewing teachers cannot apparently be dispossessed of the idea that the finest sewing is the best. On the mere ground of practical utility the notion should be discouraged; but when one thinks of all that it means in headaches, aching backs, and over-strained eyes to girls just at age when life-long health or ill-health hang in the balance, it cannot be too strongly condemned.

**A Question for Mr. Keir Hardie.**

Mr. Keir Hardie was very indignant with The Hospital a few weeks ago for publishing an article which painted the mining villages of the West of Scotland in somewhat unpleasant colours. The miners themselves, and their wives and children were said to be anything but saints either in mind or body. The *Daily Chronicle* gave a *résumé* of our article, and Mr. Keir Hardie, whilst affecting to sniff contemptuously at the original, which he declared he had not read, nevertheless made a very angry protest against our facts as reproduced by the *Chronicle*. Now The Hospital can have no conceivable reason for wishing to injure the fair fame of Scotch miners, their wives, their children, or their villages. Much less could the writer of the original article—a patriotic Scottish journalist and novelist, whose home is in the West of Scotland—be animated by any malign intents and purposes. But Mr. Keir Hardie persisted in washing his black miners and villages white with much show of virtuous indignation. Here, then, is a nut for Mr. Hardie to crack. In the *Hamilton Herald and Lanarkshire Weekly News*, of August 5th, there is published a notice of the county medical officer's annual report for the past year. The medical officer is Dr. James M'Lintock, a Bachelor of Science in Public Health, formerly medical officer of health for Bradford and Colne Valley, author of several works on sanitary subjects, and in every sense a trustworthy and experienced public official. Dr. M'Lintock states—we quote from the *Lanarkshire Weekly News*—that "he has never seen anything so uniformly bad as the condition of the miner's cottage and its surroundings in the midland counties of Scotland. . . . He blames the tenants in a certain degree for not properly attending to the cleanliness of their dwellings." Of course he blames the proprietors too, but that does not take away responsibility from the miners and their wives. On the subject of vital statistics Dr. M'Lintock reports that among the parishes "Blantyre has the unenviable position of having the highest infantile mortality, viz., 159 per thousand births, and at the same time the highest death-rate, while Bothwell ranks second in both, having an infantile death-rate of 144 per thousand births." By way of contrast, the infantile mortality at Dalziel was only at the rate of 41 per thousand births. In the middle ward 138 people died by violence, and of these 14 were children under five years old. The most frequent causes of death in these cases were drowning and burning. These are facts which cannot be denied. They are simply terrible. An infantile death-rate, in a district of country villages of 159 for every thousand born is almost incredible. Central Africa, with all its horrors, can probably not show anything half so shocking. That in one ward alone 138 people should be killed in a single year, 14 of them under five years of age, and the larger number by burning or drowning is, we would fain hope, a quite unparalleled experience even in mining districts. There must be an amount of debauchery and savagery in the West of Scotland which nothing but the most absolute and undoubted facts could make ordinary people believe, even in their dreams.

**Medical Sickness and Annuity.**

When a clergyman breaks down his income does not cease or even diminish. When the doctor cannot work his income must diminish very rapidly, and may come altogther to an end. It is a hard thing to be a poor gentleman—much harder than to be a poor workman. The sting of poverty is very keen in the flesh of the man of education. There is all the more reason, therefore, why he should, with unflinching resolution, make provision against it. At the present moment there is a society which can offer to all sorts and conditions of medical men exactly the provision they need against sickness and accident. The society, which is called the Medical Sickness, Annuity, and Life Assurance Society, is relatively rich—it has £60,000 invested. When founded a few years ago by Mr. Ernest Hart, the distinguished editor of the *British Medical Journal*, it had no invested funds at all. The sixty thousand are the result of honorary management, business capacity, and professional appreciation. Some of the cases given to illustrate the value of the society are exceedingly striking. For example: AB had paid in altogether during several years the sum of £113 3s. 3d. He has taken out, in consequence of illness, £863 2s., and he is still entitled to £2 2s. a week for twenty-three years longer. Several more cases of this kind are given in the published report for the present year. This is excellent business. Or, again, an ordinary life and annuity office pays, as a rule, 20 per cent., or much more, of its income in working expenses. The Medical Sickness and Annuity Office conducts its business at a cost of something less than 4 per cent. of its premium income. The truth is that the society is in every way an admirable institution. It is, in fact, a "Gentleman's Self-help Society" on an adequate scale, and every medical man who has no private means should hasten to claim a share of its many and great benefits.

